# A Case of Lung Adenocarcinoma With Concurrent EGFR Mutation and ALK Fusion Combined With Literature Review

**DOI:** 10.1002/ccr3.71749

**Published:** 2026-01-04

**Authors:** Yuzhu Chen, Fei Qi, Yixin Zeng, Jingwen Tan, Mingming Hu, Hongmei Zhang, Tongmei Zhang

**Affiliations:** ^1^ Department of Oncology, Beijing Chest Hospital Capital Medical University, Beijing Tuberculosis and Thoracic Tumor Research Institute Beijing China; ^2^ Laboratory for Clinical Medicine Capital Medical University Beijing China

**Keywords:** ALK tyrosine kinase inhibitor (ALK‐TKI), anaplastic lymphoma kinase (ALK) fusion, case report, EGFR mutation, EGFR‐TKI, lung adenocarcinoma

## Abstract

This case underscores the critical importance of retesting for resistant mechanisms, such as EML4‐ALK fusion, in patients with EGFR‐mutant lung adenocarcinoma who experience rapid progression on EGFR‐tyrosine kinase inhibitor (TKI) therapy. Early identification of such co‐alterations and an immediate switch to alectinib can lead to rapid and sustained clinical improvement.

## 1 Introduction

In 2022, lung cancer remained the leading cause of global cancer incidence and mortality, with approximately 2.48 million new cases and 1.8 million deaths annually [1]. Non‐small cell lung cancer (NSCLC) accounts for the majority of these cases. The discovery of driver mutations, such as in EGFR and ALK, has revolutionized the treatment paradigm for NSCLC [2]. Although EGFR mutations and ALK rearrangements are generally mutually exclusive, their concomitant presence, though rare, presents a significant therapeutic challenge due to the lack of standardized guidelines [3]. This report details a case of concurrent EGFR and ALK alterations and reviews the literature to discuss optimal management strategies.

## 2 Case Presentation

**FIGURE 1:**
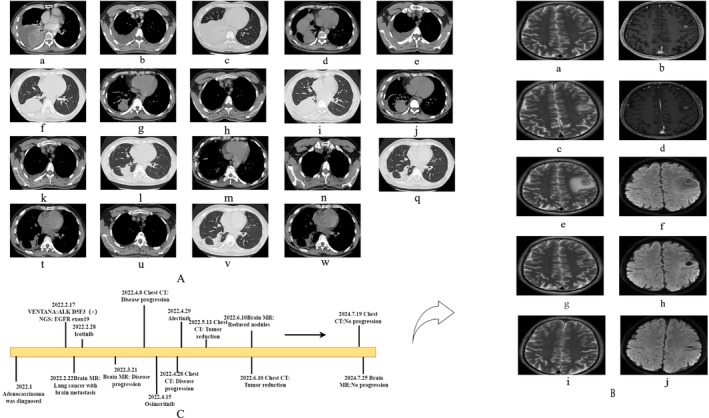
(A) It describes the changes in chest CT. (B) It describes the changes in brain MRI. (C) It is a timeline of the patient's disease progression, treatment, and outcome.

**FIGURE 2:**
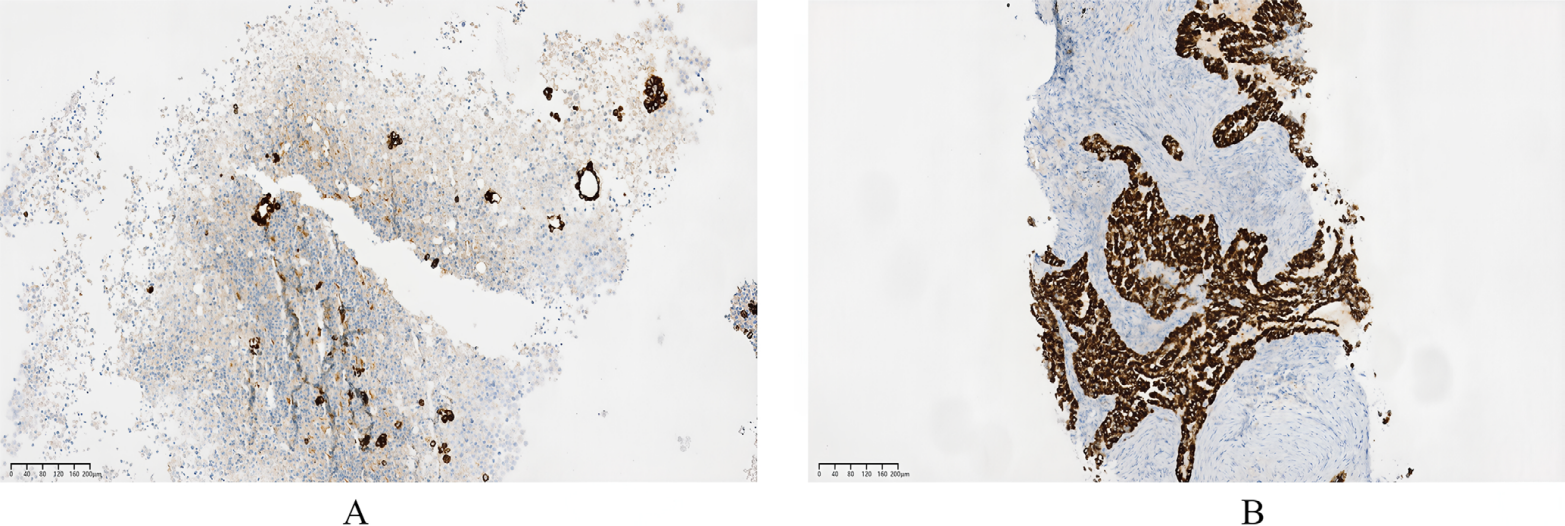
(A) Pleural effusion immunohistochemistry shows ALK fusion. (B) Lung tissue immunohistochemistry shows ALK fusion.

A 35‐year‐old male non‐smoker presented in February 2022, with a 20‐day history of chest tightness. He had no significant personal or family history of cancer. In January 2022, an external ultrasound revealed a large pleural effusion; 4400 mL of hemorrhagic fluid was drained via thoracentesis. Pleural fluid analysis showed elevated carcinoembryonic antigen (CEA), suggesting lung adenocarcinoma. A computed tomography (CT) scan confirmed a significant right pleural effusion (Figure 1A: a, b). Cytology of the effusion confirmed adenocarcinoma cells and was positive for ALK by immunohistochemistry (Figure 2A). Next‐generation sequencing (NGS) of the pleural fluid revealed an EGFR exon 19 deletion (19del) (allele frequency: 5.28%) and a low‐abundance EML4‐ALK fusion. It is critical to note that this comprehensive molecular profiling was completed prior to initiating any systemic therapy.

A repeat thoracic CT at our hospital in February showed right hilar soft tissue masses, bilateral pulmonary nodules, pleural thickening with effusion, and enlarged right supraclavicular and axillary lymph nodes (Figure 1A: c–e). A brain MRI in February identified a left frontal lobe nodule, suggestive of metastasis (Figure 1B: a,b). A biopsy of the right lower lung lesion in February confirmed adenocarcinoma and was again positive for ALK fusion (Figure 2B). The final diagnosis was stage IV lung adenocarcinoma with a concurrent EGFR exon 19 deletion and EML4‐ALK fusion.

## 3 Treatment Sequence and Rationale

### 3.1 First‐Line Therapy

In February 2022, treatment with icotinib (125 mg three times daily) was initiated. This decision was based on the confirmed EGFR sensitizing mutation and its relatively higher allele frequency, aligning with standard first‐line practice for EGFR‐mutant NSCLC at the time. Although osimertinib has become the global standard first‐line therapy for EGFR‐mutant NSCLC since the FLAURA trial, its availability and reimbursement in our region at the time were limited. Therefore, icotinib, a first‐generation EGFR‐TKI widelyaccessible and covered by insurance, was selected as the initial treatment.

### 3.2 First Disease Progression and Second‐Line Therapy

An MRI in March 2022 showed enlargement of the brain metastasis and increased edema (Figure 1B: c, d). A CT scan in April confirmed systemic progression (Figure 1A: f–h). Given the clinical and radiographic progression on icotinib, therapy was switched to osimertinib (80 mg once daily) in April 2022, as empirical escalation to a third‐generation EGFR‐TKI is a common strategy upon progression on first‐generation agents. No repeat molecular testing was performed at this time due to the rapid clinical progression and the pre‐existing knowledge of the ALK fusion, which was considered the likely resistance mechanism.

### 3.3 Second Disease Progression and Third‐Line Therapy

The patient's respiratory symptoms worsened. A CT in April showed further disease progression (Figure 1A: i–k), and an MRI indicated growth of the brain lesion (Figure 1B: e,f). In light of the rapid failure of two lines of EGFR‐TKIs and the pre‐existing molecular evidence of an EML4‐ALK fusion, a multidisciplinary team decision was made to initiate alectinib (600 mg twice daily) in April 2022, targeting the ALK pathway as the likely dominant driver of resistance. The complete treatment timeline and the efficacy are shown in Figure 1C. The rationale for switching to alectinib, rather than pursuing other EGFR‐directed strategies, was that the rapid progression on both first‐ and third‐generation EGFR‐TKIs strongly suggested that the ALK fusion was the dominant driver of resistance in this tumor. Targeting this pre‐identified ALK alteration was therefore considered the most promising therapeutic strategy.

### 3.4 Outcome and Follow‐Up

The patient's dyspnea and chest tightness improved markedly within one week of starting alectinib. A follow‐up CT in May 2022 showed significant reduction in lung lesions and pleural effusion (Figure 1A: l–n). A brain MRI in June 2022 showed a reduction in the size of the metastatic nodule and surrounding edema (Figure 1B: g,h). Subsequent imaging, including a CT in July 2024 (Figure 1A: v,w) and a cranial MRI in July 2024 (Figure 1B: i,j), demonstrated sustained partial response (PR) for over 26 months, with excellent tolerability.

## 4 Discussion

This case illustrates the therapeutic challenge and strategic decision‐making required for the rare entity of NSCLC with concomitant EGFR and ALK alterations.

### 4.1 Clarification of Treatment Rationale and Sequence

Our initial choice of first‐line icotinib was guided by the established efficacy of EGFR‐TKIs in patients with classic EGFR mutations like exon 19 del [4, 5]. The swift progression on both icotinib and osimertinib, however, suggested that the co‐existing ALK fusion was likely the primary driver of oncogenesis and resistance in this patient, a phenomenon observed in other reports [6, 7]. The subsequent dramatic and durable response to alectinib confirms this hypothesis and is consistent with the high efficacy of alectinib in ALK‐rearranged NSCLC, including its superior CNS activity [4, 5, 7].

### 4.2 Literature Comparison and Clinical Implications

As summarized in Table 1, the outcomes of patients with dual EGFR/ALK alterations are heterogeneous, but a pattern emerges. The progression‐free survival (PFS) on first‐line EGFR‐TKIs is often suboptimal and shorter than expected in pure EGFR‐mutant disease (ranging from 1 to ~13 months in reviewed cases) [4, 5, 6, 7, 8]. In contrast, the response to subsequent ALK‐TKIs can be profound and durable. For instance, Wang et al. [5] reported a PFS of 16 months on alectinib, and our patient achieved a PFS exceeding 26 months. This aligns with the findings of Zeng et al. [7] and Yin et al. [4], underscoring that ALK inhibition can be highly effective even after EGFR‐TKI failure.

**TABLE 1:** Some cases of the targeted therapies for patients with EGFR and ALK mutations reported in the last five years.

Case/reference	Sex, Age	EGFR mutation	ALK rearrangement	Targeted agents (treatment lines)	EGFR TKIs treatment progression‐free survival (PFS) (month)	EGFR TKIs adverse events (AEs)	ALK TKIs treatment PFS (month)	ALK TKIs treatment AEs	EGFR And ALK TKIs treatment PFS (month)	EGFR And ALK TKIs treatment AEs
[4]	F, 60	EGFR exons 19del/EGFR T790M	EML4‐DCTN1	Osimertinib (first) Alectinib (second)	11	Breath‐holding, headaches, dizziness, and speech	NA	NA		
[6]	F, 57	EGFR exons 19del	EML4‐ALK	Gefitinib (first) Crizotinib (second)	1	Headache and thoracodynia	NA	NA		
[7]	M, 65	EGFR exon21 L858R	ALKR3HDM1 and EML4‐ALK	gefitinib (first) Alectinib (second)	72.7	Puffy face and shortness of breath	NA	NA		
[8]	M, 38	EGFR exon21 L858R, EGFR exon 20 T790M EGFR exon 21del	STRN–ALK EML4–ALK	Gefitinib (first) crizotinib and osimertinib (second)	13	Liver and bone metastases	NA	NA		
[5]	F, 53	EGFR exons 19del	EML4‐ALK	pemetrexed plus gefitinib (first) alectinib (second)	11.2	NA	16	NA		
[9]	F, 38	EGFR L861Q	EML4‐ALK	Crizotinib (first) alectinib (second) Brigatinib (three) Alectinib and osimertinib (four)	NA	NA	13 5 6	brain metastases Myalgia increased levels of blood creatinine phosphokinases, aspartate aminotransferases, and lipases	2	
[9]	F, 42	exon 20 EGFR T790M	EML4‐ALK	alectinib (first) Lorlatinib (second) brigatinib and osimertinib (three)	NA	NA	12 2	pelvic bone Metastases lymphangitic carcinomatosis	NA	vomiting thrombopenia
This case	M, 35	EGFR exons 19del	EML4‐ALK	alectinib (first) osimertinib (second) alectinib (three)	< 1	Dyspnoea, chest tightness, progression	26	NA		

Notably, our case is distinguished by the exceptionally long PFS of over 26 months on alectinib. Several factors may have contributed to this favorable outcome: the patient's young age, good performance status, the absence of other co‐occurring resistance mutations, and the early and continuous suppression of the ALK‐driven clone. In contrast, other reported cases often involved older patients, additional resistance mechanisms, or sequential use of multiple TKIs, which may shorten PFS. Our experience reinforces that in patients with dual alterations exhibiting primary resistance to EGFR‐TKIs, ALK‐TKIs like alectinib represent a highly effective therapeutic strategy and should be considered without delay.

This case reinforces the imperative for comprehensive molecular testing at baseline. Upon rapid progression on EGFR‐directed therapy, the pre‐identified ALK fusion should be promptly targeted. Our experience, combined with the accumulating literature, suggests that for patients with dual alterations exhibiting primary resistance to EGFR‐TKIs, ALK‐TKIs like alectinib represent a highly effective therapeutic strategy and should be considered without delay.

## Author Contributions

**Yuzhu Chen:** writing – original draft, writing – review and editing. **Fei Qi:** project administration, writing – original draft, writing – review and editing. **Yixin Zeng:** writing – review and editing. **Jingwen Tan:** conceptualization, validation. **Mingming Hu:** investigation, visualization. **Hongmei Zhang:** data curation, software. **Tongmei Zhang:** conceptualization, resources, supervision.

## Funding

This work was supported by the Beijing Municipal Public Welfare Development and Reform Pilot Project for Medical Research Institutes [grant number JYY2023‐14 and JYY2023‐15] to TM Zhang.

## Ethics Statement

Ethical approval to report this case was obtained from the Ethics Committee of Beijing Chest Hospital, Capital Medical University (approval number: LW‐2024‐036).

## Consent

Written informed consent was obtained from the patient for publication of the details of their case, including all accompanying images, with assurance that anonymity would be strictly maintained.

## Conflicts of Interest

The authors declare no conflicts of interest.

## Data Availability

The authors have nothing to report.
